# Mitigating effect of L-carnitine against atrazine-induced hepatotoxicity: histopathological and biochemical analyses in albino rats

**DOI:** 10.1007/s11356-022-23568-7

**Published:** 2022-10-25

**Authors:** Walaa A. Rashad, Sara F. Saadawy, Nehal E. Refaay

**Affiliations:** 1 Faculty of Medicine, Human Anatomy & Embryology Department, Zagazig University, Zagazig, Egypt; 2 Faculty of Medicine, Medical Biochemistry Department, Zagazig University, Zagazig, Egypt

**Keywords:** Apoptosis, Atrazine, Gene expression, Hepatotoxicity, L-carnitine, Oxidative stress

## Abstract

Atrazine (ATR) is an extensively used herbicide that is often found in drinking water and waterways. After metabolization and excretion in the liver, ATR residues or its metabolites were found in tissues causing harmful effects mainly to the endocrine system and liver. This study aimed to elucidate the toxic impact of ATR on the liver and possible ameliorative effects of L-carnitine (LC). It utilized 30 adult male albino rats divided into three equal groups; the control group received 0.5 cc distilled water orally for 14 days, an ATR-treated group received ATR in a dose of 400 mg/kg BW dissolved in distilled water by oral gavage daily for 14 days, and a protected group (ATR + LC) received 400 mg/kg BW of ATR dissolved in distilled water, plus 100 mg/kg LC dissolved in distilled water by oral gavage daily for 14 days. At the end of the experiment, the liver tissue was prepared for histological and biochemical analyses and showed significant elevation of liver enzymes and oxidative parameters, altered expression of apoptotic and antiapoptotic genes, and hepatic degenerative changes in the ATR-treated group. In conclusion, atrazine induces oxidative stress, inflammation, and apoptosis in the liver of rats, and these toxic effects can be alleviated by L-carnitine.

## Introduction

Pesticides are crucial to increasing agricultural output. However, worries exist that these compounds may negatively impact both human health and the environment (Mnif et al. 2011; Gore et al. 2015). Excess use of these compounds in agricultural fields has increased their levels in the environment, thus increasing the probability of affecting physiological and biochemical performance in living organisms, especially mammals. Exposure to pesticides has been claimed to induce genotoxicity and oxidative stress in mammals (Morgan et al. 2019).

Atrazine (ATR) (2-chloro 4-ethylamine-6-isopropylamines-triazine) is a herbicide of concern because of its extensive use, endocrine disrupting abilities, and slow biodegradation (Mnif et al. 2011). It is one of the most common herbicides used in agriculture around the world and has a half-life of more than 100 days in water and 240 days in soil according to the Australian Pesticides and Veterinary Medicines Authority (APVMA 2004) and the US Environmental Protection Agency (EPA 2003), so it is exceptionally stable in the environment. As a result, ATR levels increase in soil, groundwater, and surface water over time and have a continuous impact on ecosystems (Ludlow 2010). These effects resulted in its ban in 2003 by the European Union (Commission 2004). Despite this, ATR is still used extensively in many countries (Benbrook 2016; Alonso et al. 2018), with more than 3000 and 32,500 tons administered annually in Australia and the USA, respectively (Farruggia et al. 2016; Harper et al. 2020). Additionally, ATR remains a major herbicide in Egypt due to its efficacy, low price, and widespread use among farmers (Khozimy et al. 2022).

Oral ingestion is the primary route of ATR exposure, and most likely occurs through drinking water, with concentrations typically between 0.01 and 5 mg/L (WHO 2010). Additionally, ingestion of contaminated fish is a common mean of exposure because ATR accumulates in the nervous system, gall bladder, hepatic cells, and gut of some fish. Inhalation and cutaneous penetration during application are two additional exposure routes (Rajkovic et al. 2014).

Once ATR is absorbed, it is rapidly transported to the liver where it is metabolized into deisopropyl and dealkilated (DACT) metabolites. These compounds are completely excreted in urine, along with approximately 2% of unchanged ATR. Although it undergoes biotransformation and excretion, traces of ATR and/or its metabolites have been found in some tissues (Catenacci et al. 1993), as well as in human physiological fluids, such as sperm, follicular fluid, and cervical mucus (Thornton et al. 2010).

Previous studies have recorded toxic effects of ATR, including premature and low birth weight babies (Chevrier et al. 2011), hepatotoxicity (Jin et al. 2014; Wang et al. 2017), and reproductive and immunological alterations in laboratory rodents (Pogrmic-Majkic et al. 2010). According to Song et al. (2015), ATR triggered apoptosis-related neurodegenerative damage to nerve cells as evidenced by the overexpression of *Bax*, as well as the downregulation of *Bcl-2*, *Bcl-xl*, and caspase-9. Furthermore, ATR exposure raised *Bax* and *FasL* expression and decreased *Bcl-2* expression in the kidney according to data published by Zhang et al. (2018). These data suggested that ATR caused nephrocytes to induce apoptosis.

L-carnitine (LC) (4-trimethyl ammonium 3-hydroxybutyric acid, the biologically active stereoisomer) is conditionally produced from the amino acids methionine and lysine in the brain, hepatic cells, and renal cells and is found primarily in meat and dairy products and is involved in fatty acid oxidation (Thangasamy et al. 2008). Carnitine is engaged in energy generation through fat metabolism, which is linked to oxidation and is important for delivering fatty acids into the mitochondria (Jiang et al. 2013). LC is a bioactive carnitine derivative nutrient necessary for energy generation, and a lack of it has been linked to reduced energy availability in essential organs such as the liver (Sakai et al. 2016).

Previously, Ishikawa et al. (2014) showed that LC enhanced liver function, reduced oxidative stress, and increased mitochondrial oxidation. Additionally, Alshiekh-Nasany and Douer (2016) reported that LC showed therapeutic efficacy in drug-induced hepatotoxicity. It was also reported by Vardiyan et al. (2020) that LC enhanced *Bcl-2* expression and suppressed *Bax* expression, suggesting that it may also reduce apoptosis. It was additionally found to have an antiapoptotic effect on the testicular tissue of mice receiving gamma radiation by Altun et al. (2014). They found that LC protected the apoptosis of germ cells after radiotherapy.

Therefore, the aim of the present study was to evaluate the effects of LC on histopathological changes in the hepatic tissue, antioxidant activity, and gene expression of caspase-3, *Bax*, and *Bcl-2* in ATR-induced rats.

## Materials and methods

### Chemicals

ATR is a white, odorless powder. For this study, 97% pure ATR was obtained from Kafr El Zayat Pesticides & Chemicals Co., Kafr El Zayat, Egypt. The powder was weighed and dissolved in distilled water according to the weight of the rats. LC was provided by MEPACO-MEDIFOOD Company for Pharmaceuticals and Medicinal Plants, Sharkeya, Egypt, in the form of 350 mg capsules in boxes of 20 capsules each. These capsules were opened and dissolved in distilled water.

### Animals

A total of 30 adult male albino (Sprague Dawley) rats weighing 230–250 g were obtained from the Faculty of Medicine’s animal facility, Zagazig University, for use in this study. All animals were maintained in a clean environment, fed regular meals, and provided tap water to drink. They were housed in vented polypropylene cages with stainless steel lids and wood shaving bedding, and the temperature was maintained at 23 °C ± 2 °C. They were given 15 days to acclimatize to the lab environment before the experiment began. All rats were cared for and utilized in compliance with the Zagazig University Institutional Animal Care and Use Committee’s (ZU-IACUC Committee) standard rules and regulations, with approval number *ZU-IACUC/3/F/188/2021*.

### Experimental protocol

Rats were divided into three equal groups (control group, ATR-treated group, and protected group): the control group did not receive any medication during the duration of the experiment but received 0.5 cc distilled water orally. The ATR-treated group received ATR in a dose of 400 mg/kg BW (a subacute dosage of 400 mg/kg/day (LD50/5)) dissolved in distilled water by oral gavage daily for 14 days (Juliani et al. 2008), and the protected group rats were given 400 mg/kg BW of ATR dissolved in distilled water, plus 100 mg/kg LC dissolved in distilled water, by oral gavage daily (Abd‐Elrazek and Ahmed‐Farid 2018) for 14 days. The rats were observed for overall behavior, toxicity signs, and mortality throughout the study.

### Sample preparation

At the end of the experiment, rats were weighed, anesthetized with an intra-peritoneal injection of 30 mg/kg thiopental, and then sacrificed. A laparotomy was conducted to expose the liver. To eliminate all red blood cells and clots, tissues were extensively perfused with a 50 mM sodium phosphate buffer saline (100 mM Na_2_HPO_4_/NaH_2_PO_4_) (pH 7.4) and 0.1 m ethylenediaminetetraacetic acid (EDTA) solution prior to dissection. Then, the harvested liver of each rat was divided into three parts: a small part for gene expression analysis, another part for homogenate tissue analyses, and the remaining part for histopathological examination.

### Serum biochemical analysis

The rats were sedated, and blood samples were taken from the retro-orbital venous plexus using anticoagulated micro-capillary syringes, which were allowed to coagulate at ambient temperature for 30 min before being processed at 3000 rpm. Pure sera which had not been hemolyzed were quickly obtained and processed at − 20 °C for biochemical analysis. Alanine aminotransferase (ALT) and aspartate aminotransferase (AST) activities were measured using the pyruvate and oxaloacetate concentration monitoring methods, respectively (Reitman and Frankel 1957). Diamond Diagnostics Chemical Company reagent kits were used for the assays (Cairo, Egypt).

### Homogenate tissue analysis for oxidative stress parameters

Liver tissues were homogenized in 10 × phosphate-buffered saline (PBS), pH 7.4, containing 0.2 g potassium chloride (KCl), 8 g sodium chloride (NaCl), 0.24 g potassium dihydrogen phosphate (KH_2_PO_4_), and 1.4 g disodium hydrogen phosphate (Na_2_HPO_4_), and then centrifuged at 10,000 g for 15 min at 4 °C. The collected supernatant was used to assay the activity of the following: superoxide dismutase (SOD), an antioxidant enzyme that was assayed according to the method of Marklund and Marklund (1974). The difference in color absorbance at 430 nm at 0 and 10 min was assessed by measuring enzyme activity using a biodiagnostic kit (SD 25 21, bio-diagnostic.com, Giza, Egypt). Malondialdehyde (MDA): Liver oxidative stress marker was measured by the thiobarbituric acid method (Liu et al. 1997), using a biodiagnostic kit (MD 2529, bio-diagnostic.com, Giza, Egypt).

### RNA isolation and semi-quantitative reverse transcriptase-PCR (RT-PCR)

Total RNA was extracted from 30 mg of rat liver using Trizol (Invitrogen; Thermo Fisher Scientific, Inc., Waltham, MA, USA), the efficiency and density of the RNA were measured using the NanoDrop® ND-1000 Spectrophotometer (NanoDrop Technologies, Wilmington, DE, USA), and cDNA was generated with a HiSenScriptTM RH ( −) cDNA Synthesis Kit (NanoDrop Technologies, Wilmington, DE, USA). The real-time RT-PCR was performed in a Mx3005P Real-Time PCR System (Agilent Stratagene, Santa Clara, CA, USA) using TOPreal™ qPCR 2X PreMIX (SYBR Green with low ROX) (Cat. #P725 or P750, Enzynomics, Daejeon, Korea) according to the manufacturer’s criteria.

Denaturation at 95 °C for 12 min was followed by 40 cycles of denaturation at 95 °C for 20 s, annealing at 60 °C for 30 s, and extension at 72 °C for 30 s under PCR cycling conditions. Sangon Biotech (Beijing, China) synthesized the oligonucleotide-specific primers listed in Table 1 (Khamis et al. 2021). Following PCR amplification, a dissolving curve analysis was performed. The mRNA expression of a well-known housekeeping gene, *GAPDH*, was utilized to equalize the target genes’ expression levels. The results were expressed as fold-changes and compared with the control group using the 2^−ΔΔC^_T_ method (Livak and Schmittgen 2001).

**Table 1:** Primers

	Forward primer (5′–3′)	Reverse primer (5′–3′)	Size	Accession no
Bax	CGAATTGGCGATGAACTGGA	CAAACATGTCAGCTGCCACAC	109	NM_017059.2
Bcl-2	GACTGAGTACCTGAACCGGCATC	CTGAGCAGCGTCTTCAGAGACA	135	NM_016993.1
Casp-3	GAGACAGACAGTGGAACTGACGATG	GGCGCAAAGTGACTGGATGA	147	NM_012922.2
Gapdh (Rat)	GGCACAGTCAAGGCTGAGAATG	ATGGTGGTGAAGACGCCAGTA	143	NM_017008.4

Khamis et al. (2021)

### Histological and morphometric examination

After dissection, liver tissues were collected from the rats and rinsed with saline, and then specimens were extended on filter paper and preserved in buffered formalin at a concentration of 10%, pH 7.4. The fixed specimens were sliced, processed, and embedded in paraffin blocks which were then cut into 4-μm paraffin sections. These were stained with hematoxylin and eosin, Masson trichrome, and toluidine blue stains (Bancroft and Gamble 2008). The slides were examined and photographed using a light microscope (LEICA ICC50 W, Leica Microsystems, Wetzlar, Germany), in the Anatomy Department, Faculty of Medicine, Zagazig University.

The area percentage of collagen fibers from Masson’s trichrome-stained liver sections was measured at 400 × magnification using image analysis software (ImageJ 1.36b, http://rsbweb.nih.gov/ij). Quantitative data was estimated in five different, non-intersected sections, for the same slide of each animal. This resulted in a total of 25 fields for each group.

### Immunohistochemical and morphometrical examination

After liver tissue Sections. (5 μm) were deparaffinized, endogenous peroxidase was satisfied with 3% H_2_O_2_/methanol for 5 min. Primary antibodies were applied to sections overnight at 4 °C, followed by a 1-h incubation at room temperature with a biotinylated secondary antibody. By treating the slices in diaminobenzidine, antibody binding was seen. PBS was used for all the incubations, and then the sections were dehydrated and coated after being counterstained with hematoxylin. The slices were then incubated with a rabbit polyclonal anti-GFAP (glial fibrillary acidic protein) primary antibody (1:1000, 4 °C, overnight; Agilent Dako, Santa Clara, CA, USA) and then with a biotinylated goat anti-rabbit secondary antibody (Agilent Dako).

The area percentage of GFAP immunoreactivity at 400 × magnification was measured using image processing software (ImageJ 1.36b, http://rsbweb.nih.gov/ij). Quantitative data was estimated in five different non-intersected sections for the same slide of each animal. This resulted in a total of 25 fields for each group.

#### Statistical analysis

The Statistical Package for Social Science (SPSS for Windows, version 18.0, SPSS, Inc., Chicago, IL, USA) was used to statistically analyze the biochemical and morphometric data collected. The normal distribution of the data was assured using the Shapiro–Wilk test, where *p* > 0.05. For ease of presentation, normally distributed data were summarized by mean and standard deviation. As a key assumption of the one-way ANOVA, homogeneity of variance was assessed among the compared groups using Bartlett’s test for equality of variances, where *p* > 0.05. The mean values of individual groups were compared using the one-way ANOVA test. Multiple comparisons were assessed using the least significant difference test. A value of *p* < 0.001 was statistically significant, while *p* > 0.05 was considered non-statistically significant.

## Results

ATR-treated animals displayed decreased weights with changes in eye color and thinned hair; while the administration of LC with ATR reduced the weight decrease somewhat.

### Biochemical results

#### Serum enzymes and oxidative parameters

As illustrated in Table 2, the ATR-treated group showed a significant increase in AST and ALT levels compared with the control, recording percentage increases of 96.1% and 99.3%, respectively. In contrast, the protected group showed significant decreases of AST and ALT compared with the ATR-treated group, with changes of 18.9% and 33.1%, respectively. Concerning oxidative parameters, MDA levels in the treated group were considerably greater than in the control group, with a 74.9% change, suggesting oxidative damage. Using LC in the protected group led to a significant decrease (28.5%) in MDA levels compared with the ATR-treated group, indicating decreased lipid peroxidation, SOD recorded a decrease of 37.3% in the ATR-treated group compared with the control, and the percentage increased 28% in the protected group when compared with the ATR-treated group.

**Table 2:** Serum levels of liver enzymes and oxidative stress markers

Parameter	Control *N* = 10 Mean ± sd	ATR treated *N* = 10 Mean ± sd	% of change	Protected *N* = 10 Mean ± sd	% of change	*P* value
AST (U/l)	23.1 ± 2.3	45.3 ± 4.7ª	+ 96.1%	36.7 ± 3.5^a,b^	− 18.9%	< 0.001*
ALT (U/l)	14.7 ± 1.7	29.3 ± 4.1ª	+ 99.3%	19.6 ± 1.8^a,b^	− 33.1%	< 0.001*
MDA (nmol/ml)	28.1 ± 2.38	49.16 ± 3.2ª	+ 74.9%	37.57 ± 2.6^a,b^	− 28.5%	< 0.001*
SOD (u/ml)	20.57 ± 2.57	12.78 ± 2.29^a^	* − *37.3%	16.57 ± 2.7^a,b^	+ 28%	< 0.001*

One-way ANOVA

*Sd* standard deviation

*NS* non-significant

^*^Significant (*P* < 0.001)

^a^Versus control

^b^Versus treated

*AST* aspartate aminotransferase, *ALT* alanine aminotransferase, *MDA* malondialdehyde, *SOD* superoxide dismutase, *N* number

% of change were calculated by comparing the ATR-treated group with control and protected group

#### The real-time quantitative PCR

It was demonstrated that the antiapoptotic *Bcl-2* gene expression ratio was 0.228 ± 0.03 and 0.858 ± 0.07-fold in the ATR-treated and ATR-protected groups, respectively. Furthermore, the proportion for *Bax*, a pro-apoptotic gene, was 4.31% ± 0.53% in the ATR-treated rats and 1.87% ± 0.29% in the control rats (Table 3).

**Table 3:** Effect of ATR and LC on gene expression of apoptotic pathway genes

Parameter	Control *N* = 10	ATR treated *N* = 10 Mean ± sd	Protected *N* = 10 Mean ± sd	*P* value
Bax	1.0	4.31 ± 0.53ª	1.87 ± 0.29^a,b^	< 0.001*
Bcl-2	1.0	0.228 ± 0.03	0.858 ± 0.07^b^	< 0.001*
Caspase-3	1.0	6.14 ± 1.07ª	2.88 ± 0.47^a,b^	< 0.001*
Bax/Bcl-2 Ratio	1.0	19.5 ± 4.5	2.2 ± 0.47	< 0.001*

One-way ANOVA

*Sd* standard deviation

*NS* non-significant

^*^Significant(*P* < 0.001)

^a^Versus control

^b^Versus treated

*N* number

The expression of *Bcl-2* significantly differed between the experimental groups, and when compared with the ATR-treated group, the transcription of this gene was greater in the protected group (*p* > 0.001). Furthermore, compared with the protected and control groups, the *Bax* signaling pathway was considerably greater in the treated group (*p* > 0.001). Additionally, compared with the other two groups, *caspase-3* genetic variation was considerably higher in the ATR-treated group (*p* > 0.001). The proportion of *Bax* to *Bcl-2* (*Bax*/*Bcl-2*) was computed as 1.0, 19.5 ± 4.5, and 2.2 ± 0.47 for the control, treated, and protected groups, respectively.

### Histological results

#### Hematoxylin and eosin

**Fig. 1:**
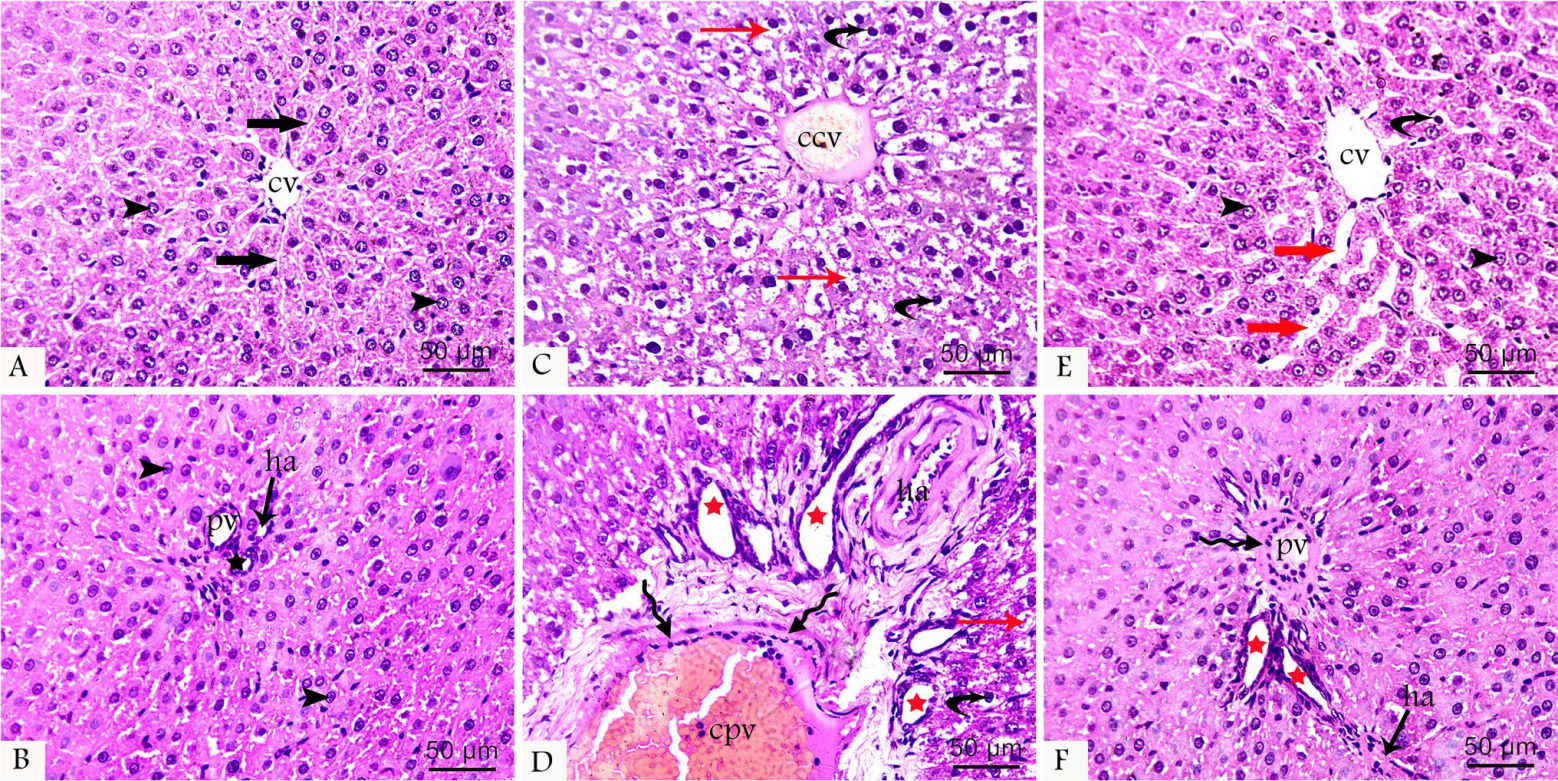
H&E-stained liver tissue sections at 400 × magnifications from all groups showing normal hepatocytes (arrowhead) arranged in cords radiating from the central vein (cv) and entrapping normal blood sinusoids (black thick arrow) between the cords. Components of the portal tract, portal vein (pv), hepatic artery (ha), and bile duct (black star) appear normal in control group (**A** and **B**). ATR-treated group (**C** and **D**) shows dilated congested central (ccv) and portal (cpv) veins, pyknotic nuclei (curved arrow), and vacuolated cytoplasm (red arrow) of hepatocytes, fibrosis (wavy arrow) around the portal vein, and formation of new bile ductules (red star). The protected group (**E** and **F**) shows restoration of the normal liver structure with slight dilatation of the central (cv) and portal (pv) veins and hepatic sinusoids (thick red arrow), pyknotic nuclei (curved arrow) of hepatocytes, fibrosis (wavy arrow) around the portal vein, and new bile ductule (red star)

Histological examination of tissue sections from the control group displayed normal histological organization of the liver. This consisted of hexagonal hepatic lobules, with each lobule consisting of cords of hepatocytes radiating from the central vein toward the periphery and entrapped liver blood sinusoids between them. Hepatocytes were polygonal-shaped cells containing vesicular nuclei and prominent nucleoli with acidophilic cytoplasm. The portal tract was found at the periphery with its three components, branches of the portal vein, hepatic artery, and bile duct. In the ATR-treated group, the normal liver structure was disturbed, and degenerative changes were observed in the hepatocytes in the form of faint pyknotic nuclei that were sometimes even absent and vacuolation of their cytoplasm. Dilated congested central veins and widened congested liver sinusoids were also observed. Edema of the portal tract, formation of new bile ductules, and dilated congested portal veins with a surrounding fibrotic area were also observed. In the protected group, the histopathological organization of the liver was restored to some extent with the restoration of normal liver structures but still remained minimal affection in the form of mild dilation of the central and portal veins as well as hepatic sinusoids, hepatocyte degeneration, fibrosis around the portal vein, and new bile ductules (Fig. 1).

#### Masson’s trichrome

**Fig. 2:**
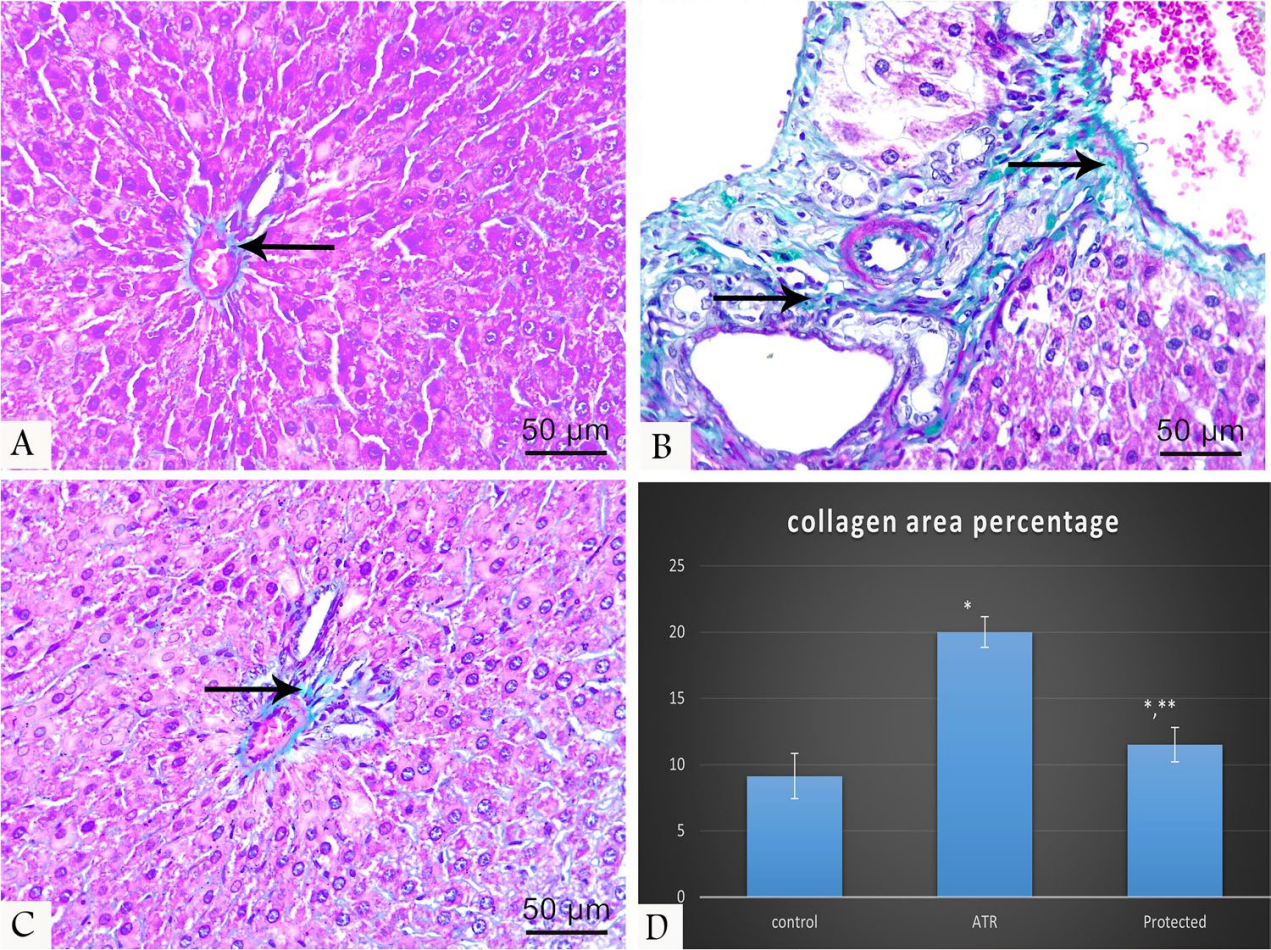
Masson’s trichrome-stained liver tissue sections at 400 × magnifications showing the distribution of collagen fibers (arrow) around the portal tract which is normally distributed in the control group (**A**), markedly increased around the vessels in ATR-treated group (**B**), and slightly increased in the protected group (**C**). Chart shows morphometrical analysis of area percentage of collagen fibers (**D**)

Normal distribution of collagen fibers was observed in the control group, while the ATR-treated group displayed an apparently increased amount of collagen fibers, mostly around blood vessels in the portal tract. In the protected group, a slight increase of collagen fiber distribution was observed (Fig. 2).

#### Toluidine blue

**Fig. 3:**
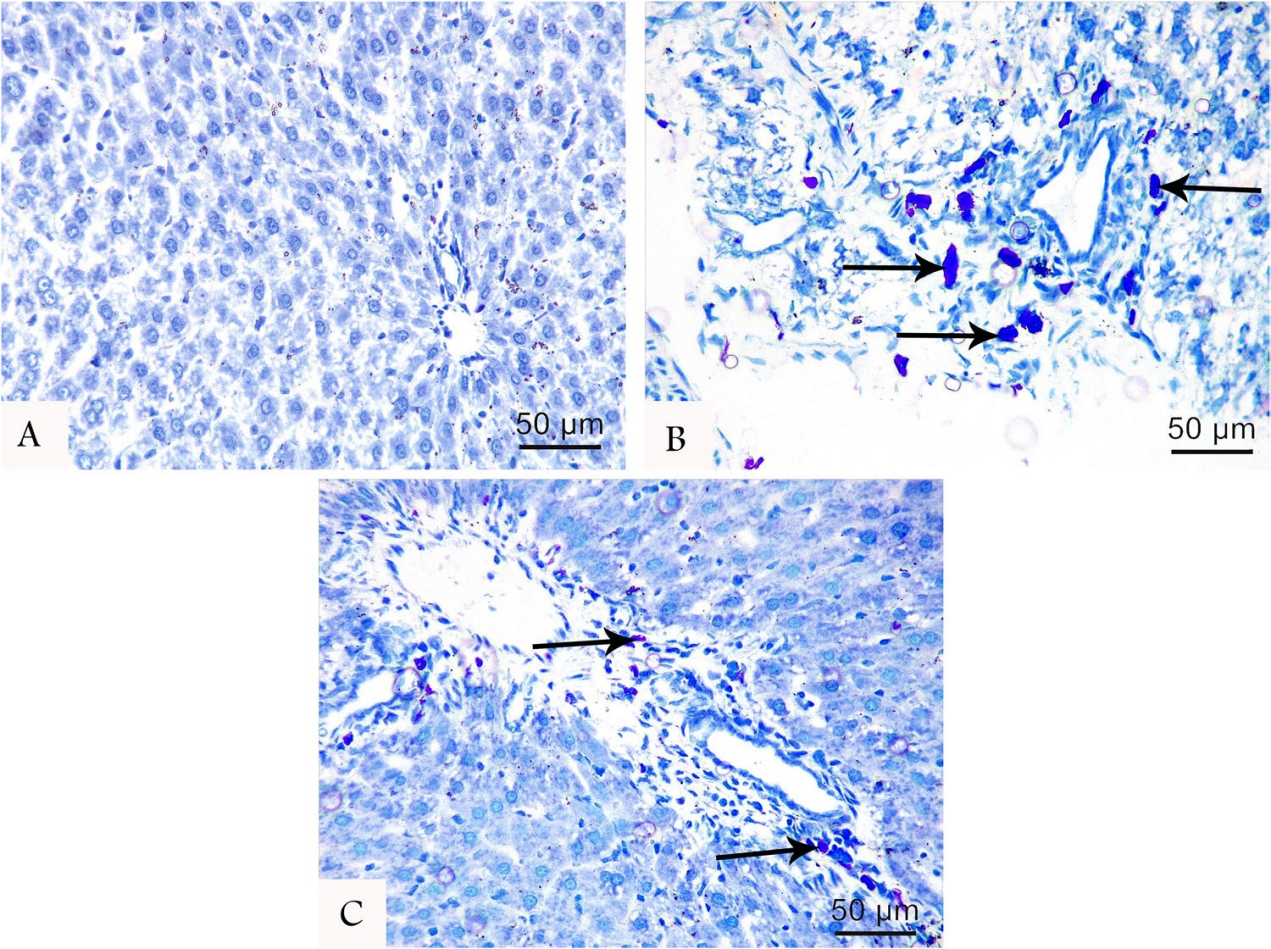
No mast cells were noticed in the control group (**A**). Toluidine blue-stained liver tissue sections at 400 × magnifications showing heavy infiltration of mast cells (arrow) around the portal tract in the ATR group (**B**) compared with protected groups (**C**)

Toluidine blue-stained liver tissue sections displayed heavy infiltration of polymorphic mast cells around the portal tract in ATR-treated group. In the protected group, some cells were also noticed, but no cells were observed in the control group (Fig. 3).

### Immunohistochemical results

**Fig. 4:**
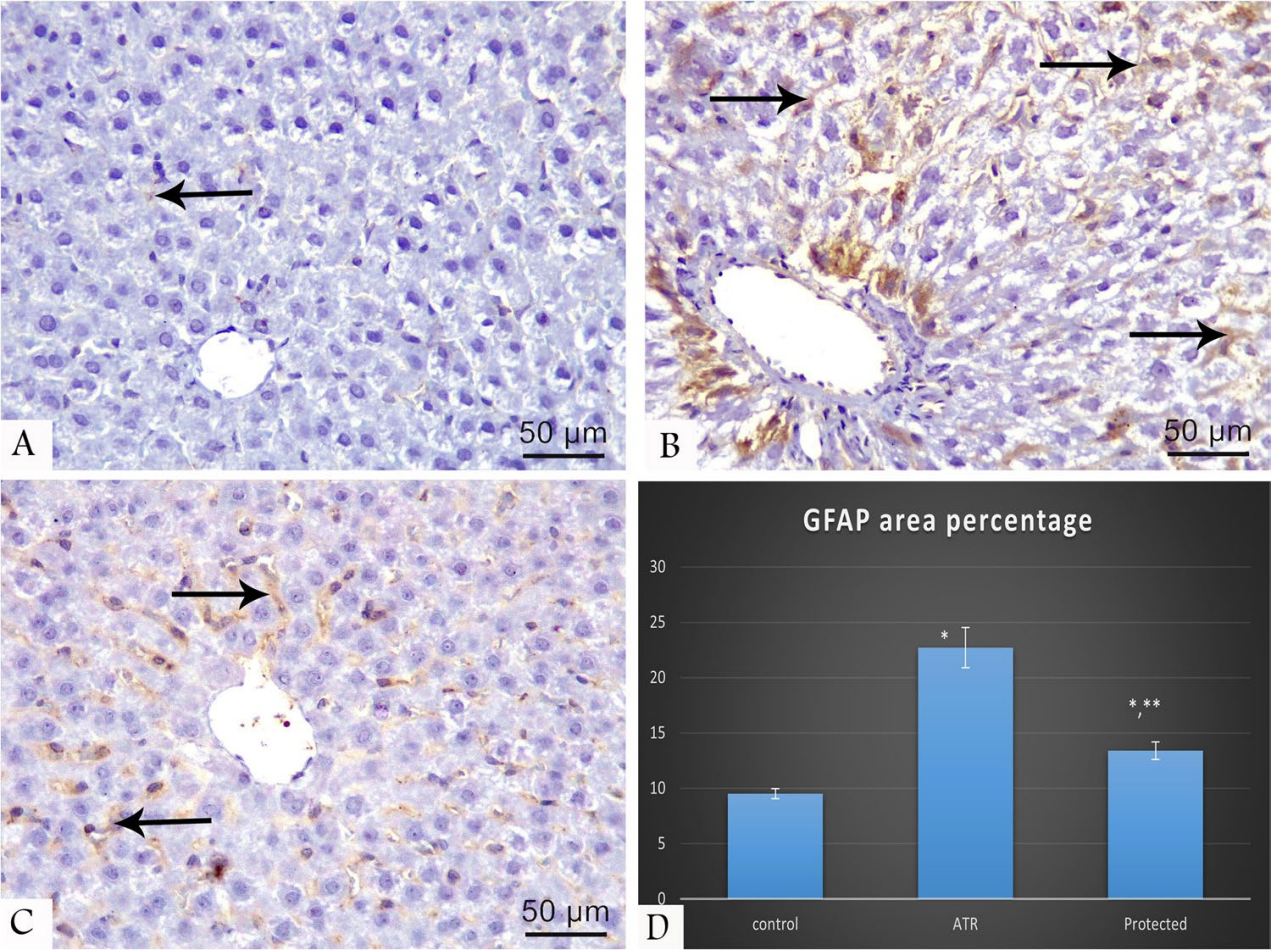
GFAP-immunostained liver tissue sections at 400 × magnifications showing positive reaction (arrow) which is minimal in the control group (**A**), marked in ATR-treated group (**B**), and moderate in the protected group (**C**). Chart shows morphometrical analysis of GFAP positive stained area percentage (**D**)

GFAP-immunostained liver tissue sections displayed a marked increase of immunoreactivity in the ATR-treated group compared with the protected group, while minimal reaction was detected in the control group (Fig. 4).

### Morphometrical results

Area (%) of collagen fibers and GFAP immunoreactivity in the ATR-treated group showed a statistically significant difference compared with the control (Table 4) and protected groups (Figs. 2D and 4D).

**Table 4:** Percentage of collagen fibers and area stained positive with GFAP% of different studied group

Parameter	Control group *N* = 10 Mean ± sd	ATR treated *N* = 10 Mean ± sd	Protected *N* = 10 Mean ± sd	*P* value
Collagen fibers area percentage	9.13 ± 1.7	20 ± 1.15^a^	11.5 ± 1.3^a,b^	< 0.001^a*^
GFAP percentage area	9.5 ± 0.46	22.7 ± 1.8^a^	13.4 ± 0.79^a,b^	< 0.001^a^^*^ < 0.001^b^^*^

One-way ANOVA

*Sd* standard deviation

*NS* non-significant (*P* > 0.05)

^*^Significant (*P* < 0.05)

^a^Versus control

^b^Versus treated

*N* number

## Discussion

ATR is a widespread herbicide used to promote growth in maize, pineapple, sorghum, sugar beet, and cereal crops. It has a half-life of 95–350 days and is not decayed. For many years, ATR residues, its metabolite deethylatrazine, and other metabolites have polluted soil and water (Singh et al. 2008). Therefore, ATR poisoning is inevitably causing neurologic, immunological, and cardiac dysfunction, skin-related diseases, and respiratory problems (Jestadi et al. 2014). Moreover, adult ATR abuse has been associated with various non-lymphoma cancers, and premature birth and intrauterine growth retardation have been correlated with the presence of ATR in drinking water. The glandular (hormonal) system and the liver are the primary targets of ATR in humans and livestock (Abarikwu 2014).

In the present study, the ATR-treated group had significantly higher AST and ALT levels compared with the control group and protected groups. This contrasted with findings of Campos-Pereira et al. (2012), who found no considerable increase in serum ALT levels, implying that the necrotic zone observed in the histologic examination was insufficient to induce a significant increase in ALT levels. However, our findings were consistent with those of Jestadi et al. (2014), who found a significant increase in AST and ALT activity in rats treated with ATR (300 mg kg/BW) compared with the control group. The current study’s observed increase in ALT was particularly associated with ATR-induced liver cell damage (Konstantinova and Russanov 1999). The increased serum AST is thought to be related to ATR-induced mitochondrial damage caused by reactive oxygen species (ROS) (Zilva et al. 1988).

In this study, signs of necrosis were seen as weak pyknotic nuclei, which were also seen in rats given chlorpyrifos for 4 weeks (Ezzi et al. 2016). According to Campos-Pereira et al. (2012), necrosis is caused by cellular breakdown, which is characterized by organelle swelling and disturbed cytoplasm, followed by nuclei shrinkage and collapse. To confirm these results, the mechanism of ATR-induced cell death was investigated. Two genes known to participate in apoptosis are *Bcl-2* and *Bax*; these genes are either actively or passively associated with ROS metabolism (Ye et al. 1999) and are also required for the induction of apoptosis (Polyak et al. 1997). The present study evaluated the impacts of ATR and LC on the expression of *Bcl-2* and *Bax*, and the outcomes of this experiment showed that the expression of *Bax* in ATR-treated rats was significantly increased compared with the control group, while the expression of *Bcl-2* in ATR-treated rats was significantly decreased compared with the control group. These results confirm that ATR enhances the expression of apoptotic genes and accelerates the process of apoptosis, eventually leading to impaired liver function**.** Moreover, the changes in *Bax* and *Bcl-2* expression result in a high *Bax*/*Bcl-2* ratio, which is a significant factor in determining cell vulnerability to apoptosis. Thus, ATR-induced apoptosis is regulated by a harmony of apoptosis-enhancing and apoptosis-inhibiting molecules.

Additionally, caspase-3 gene expression was found to be significantly increased in the ATR-only treated group. This was confirmed by similar observations by Abarikwu and Farombi (2015), who investigated the effects of ATR on human neuroblastoma (SHSY5Y) cells, and Morgan et al. (2019), who evaluated the modulatory role of glycyrrhizic acid susceptibility against harmful impacts of ATR in the rabbit spleen.

SOD, CAT, and GPx are antioxidant enzymes that guard against peroxidation by transforming free radicals or reactive oxygen intermediate products to non-radical products (Montilla et al. 1998). SOD acts as an initial protective barrier against oxygen-derived oxidative stress, reducing cell damage through O_2_^−^ dismutation (Fujii et al. 2003). In this study, it was discovered that there was a considerable rise in SOD activity in ATR-treated rats when compared with non-treated rats. The elevation in SOD activity following ATR treatment seems to be an adaptation in response to the enhanced production of free radicals. Animals exposed to xenobiotics have been shown to have increased SOD activity in a variety of tissues (Wafa et al. 2013). In contrast, other studies observed that elevated SOD levels in the hepatic tissue of ATR-exposed rats were not statistically significant when compared with control rats (Jestadi et al. 2014).

MDA is an oxidative stress marker that can be used to assess the redox balance of healthy and damaged tissues (Marrocco et al. 2017). In this study, exposure to ATR caused an increase in MDA production that was significantly higher in ATR-treated and protected groups compared with control animals, thus indicating lipid peroxidation. Despite this finding, using LC in the protected group led to a significant decrease in MDA level compared with the toxic level in the ATR-only treated group, indicating decreased lipid peroxidation, which was consistent with the studies conducted by Nwani et al. (2010), Bhatti et al. (2011), and Campos-Pereira et al. (2012).

Histological results in this work revealed histological alterations in ATR-treated hepatocytes in the form of cellular degenerative changes and vacuolization. These findings were consistent with those of Campos-Pereira et al. (2012), who administered an ATR aqueous suspension at a dosage of 400 mg/kg/day orally to mature male Wister rats for 14 days. These effects were also noted in mice treated with 200 mg ATR for 28 days (Batool et al. 2021). The accumulation of lipids in cytoplasmic vesicles is known as cytoplasmic vacuolation, but it has also been proposed that vacuolar production is a defensive cellular response to toxic substances designed to reduce cellular metabolism (Bourne 2012).

In the current study, engorged central veins and enlarged congested liver sinusoids were observed. This concurred with Lin et al. (2016), who discovered central vein hyperemia, as well as hepatic sinusoids in quail exposed to different dosages of ATR, with increasing damage as the dosages increased. Furthermore, Sena et al. (2021) discovered ballooning and congestion of liver cells in frog livers exposed to ATR, with exacerbation of the damage with higher ATR concentrations.

Using toluidine blue staining, the current study discovered increased mast cell infiltration around the portal tract after ATR treatment that copes with Mizota and Ueda (2006) who reported that ATR induces some inflammatory effects mediated by mast cell degranulation that may serve as additional warnings of the ecological problems. Also, it was consistent with the findings of Deshmukh and Ramteke (2015), who discovered prolonged intercellular inflammation, lymphocytes, and eosinophil invasion in rats given ATR for 120 days. This could be due to oversensitivity to ATR or inflammatory responses.

In this study, many collagen fibers were detected around the portal zone identified by Masson’s trichrome stain in ATR-only treated rats. This may be related to the observed inflammatory cellular penetration. Morphometric data verified this observation, revealing a considerable rise in collagen area percentage in the ATR group compared with the control group. This could result from mitochondrial dysfunction and weakness caused by ROS and lipid peroxidation agents, which can result in cell apoptosis and necrosis, as well as amplification of a pathway contributing to fibrosis and collagen production (Duval et al. 2014).

ATR-induced tissue toxicity is caused by a multitude of mechanisms. It may result in an increase in ROS, causing oxidative stress and compromising energy metabolism (Thompson and Al-Hasan 2012). An elevation in the expression of *Slc27a5*, which codes for the fatty acid transporter protein 5, was also linked to ATR. This gene is present solely in the liver and is implicated in fatty acid intake, which can lead to the formation of triglycerides in the organ (Gimeno 2007). There were also increases in low-density lipoprotein receptor expression in embryos exposed to ATR prenatally, a protein that transports low-density lipoproteins into liver cells (Harper et al. 2020).

Hepatic stellate cells (HSC) (perisinusoidal cells) are type of cells that serve as the foundation for GFAP immunohistochemical detection. Expression of GFAP was reported in quiescent stellate cells in vivo, with an increased expression in the acute response to injury in rats and a downregulation in chronic cases (Morini et al. 2005). This study’s ATR-treated group had much higher immunological expression of the HSC marker GFAP than the control and protected groups. According to some researchers, GFAP development in the liver is associated with hepatic fibrosis and inflammatory cellular invasion, since stimulated HSC release cytokines which drive inflammatory cells. Additionally, several authors have found that HSC stimulation is linked to the involvement of ROS and oxidative stress in promoting the development of proinflammatory and profibrotic molecules (Hassan et al. 2018).

L-carnitine (LC) is a conditionally required amino acid that is synthesized in kidneys, the brain, and hepatocytes from the amino acids methionine and lysine but is mostly obtained through food (Cave et al. 2008). It transports long-chain fatty acids across the mitochondrial membrane, permitting the discharge of oxidative energy (Hassan et al. 2015; Gao et al. 2017). Since it is produced in the liver and has a role in the transfer of fatty acids, it has been employed frequently in investigations of liver fibrosis (Demiroren et al. 2014). This study showed that LC has both protective and therapeutic effects on AST and ALT, as the results showed a decrease in ALT and AST levels in the protected group compared with the ATR-treated group. This was in agreement with Karabulut et al. (2021), who recommended the use of LC to prevent the process of irreversible liver damage.

Additionally, LC guards against oxygen-free radical damage to DNA and cell membranes (Makker et al. 2009). The study also found that the group protected by LC had higher *Bcl-2* expression and lower *Bax* expression. This finding implies that LC can decrease the harmful effects of ATR and decrease the production of genes that promote apoptosis, such as *Bax*. This finding concurs with Cankorkmaz et al. (2009), who hypothesized that LC would speed up the healing of testicular damage in mice by lowering apoptosis. Additionally, LC prevented the apoptosis of germ cells during radiation therapy, according to Altun et al. (2014), who studied the antiapoptotic impact of the compound on the testicular tissue of mice exposed to gamma radiation.

Finally, this study discovered that LC contributed to the restoration of normal histological structure in the liver, with minor dilation of the central and portal veins, hepatic sinusoids, and hepatocyte degeneration, and enhanced antioxidant enzymes. These findings concurred with Koohpeyma et al. (2021), who found that LC decreased oxidative stress, kidney morphological alterations, and the probability of apoptosis generated by monosodium glutamate.

## Conclusion

Exposure to ATR resulted in detrimental functional and structural alterations in the livers of albino rats. Significant elevation of liver enzymes and oxidative parameters, altered expression of apoptotic and antiapoptotic genes, hepatic degenerative changes, and strong immunoreactivity to GFAP were all indicative of the oxidative, inflammatory, and apoptotic mechanisms mediated by ATR. LC demonstrated a noticeable ameliorative effect on ATR-induced hepatotoxicity, mostly via potent antioxidant, anti-inflammatory, and antiapoptotic properties.

## Data Availability

The datasets generated and analyzed during the current study are available from the corresponding author on reasonable request.
